# Drinking and smoking polygenic risk is associated with childhood and early-adulthood psychiatric and behavioral traits independently of substance use and psychiatric genetic risk

**DOI:** 10.1038/s41398-021-01713-z

**Published:** 2021-11-13

**Authors:** Flavio De Angelis, Frank R. Wendt, Gita A. Pathak, Daniel S. Tylee, Aranyak Goswami, Joel Gelernter, Renato Polimanti

**Affiliations:** 1 Department of Psychiatry, Yale University School of Medicine, West Haven, CT USA; 2 Veteran Affairs Connecticut Healthcare System, West Haven, CT USA

**Keywords:** Genomics, Pathogenesis

## Abstract

Alcohol drinking and tobacco smoking are hazardous behaviors associated with a wide range of adverse health outcomes. In this study, we explored the association of polygenic risk scores (PRS) related to drinks per week, age of smoking initiation, smoking initiation, cigarettes per day, and smoking cessation with 433 psychiatric and behavioral traits in 4498 children and young adults (aged 8–21) of European ancestry from the Philadelphia neurodevelopmental cohort. After applying a false discovery rate multiple testing correction accounting for the number of PRS and traits tested, we identified 36 associations related to psychotic symptoms, emotion and age recognition social competencies, verbal reasoning, anxiety-related traits, parents’ education, and substance use. These associations were independent of the genetic correlations among the alcohol-drinking and tobacco-smoking traits and those with cognitive performance, educational attainment, risk-taking behaviors, and psychopathology. The removal of participants endorsing substance use did not affect the associations of each PRS with psychiatric and behavioral traits identified as significant in the discovery analyses. Gene-ontology enrichment analyses identified several neurobiological processes underlying mechanisms of the PRS associations we report. In conclusion, we provide novel insights into the genetic overlap of smoking and drinking behaviors in children and young adults, highlighting their independence from psychopathology and substance use.

## Introduction

Alcohol drinking and tobacco smoking may result in direct or indirect health concerns. Psychoactive compounds such as ethanol and nicotine act primarily on mental processes and therefore can affect mood, feelings, and behavior [1], but they are also related to many negative health outcomes [2–4]. Drinking and smoking represent two of the three leading preventable causes of death in the US [5]. Understanding the molecular and behavioral processes underlying the predisposition to alcohol drinking and tobacco smoking could lead to better strategies aiming to prevent the cascade of psychiatric and behavioral impairments associated with problematic drinking and smoking. Large-scale genome-wide association studies (GWAS) of traits related to alcohol drinking and tobacco smoking demonstrated that the predisposition to these complex behavioral traits is highly polygenic (i.e., thousands of variants with small effects) [6–12]. To date, GSCAN (GWAS & Sequencing Consortium of Alcohol and Nicotine) has completed the largest genome-wide meta-analysis across multiple drinking and smoking behaviors short of dependence on either of these substances, analyzing up to 1.2 million individuals [13]. GSCAN investigated one alcohol-drinking phenotype (drinks per week, DPW) and four tobacco-smoking phenotypes. These included cigarettes per day (CPD, average number of cigarettes smoked per day), smoking initiation (SI, smoker versus non-smoker), smoking cessation (SC, current versus former smoker), and age of smoking initiation (ASI, age at which an individual started smoking regularly). While ASI is negatively genetically correlated with all the other traits (from rg = −0.10 with respect to DPW to rg = −0.71 for SI), DPW and the other smoking phenotypes share positive genetic correlations ranging from rg = 0.07 (CPD vs. DPW) to rg = 0.42 (SC vs. CPD). These traits also showed a broad spectrum of genetic correlations including behavioral traits (e.g., risk tolerance and neuroticism), psychiatric disorder (e.g., major depressive disorder and schizophrenia), and physical health outcomes (e.g., obesity and coronary artery disease) [13]. Due to the large effects of tobacco and alcohol on human health, it is challenging and important to distinguish whether the genetic correlations observed are due to the consequences of alcohol drinking and tobacco smoking or to the genetic etiology shared between these traits and other complex phenotypes. To dissect the pleiotropic mechanisms related to alcohol drinking and tobacco smoking, we investigated their genetic liability through the polygenetic risk scores (PRS) derived from GSCAN genome-wide association data with respect to psychiatric and behavioral traits assessed in the Philadelphia Neurodevelopmental Cohort (PNC). Similar to other studies [14–20], we used a high-resolution phenome-wide screening approach investigating hundreds of traits related to different neurodevelopmental domains. Due to the limited alcohol and tobacco use in the PNC participants, testing the association of genetic liability to alcohol drinking and tobacco smoking with a wide range of elements of psychiatric and behavioral assessment can permit us to generate novel hypotheses regarding the mechanisms leading to smoking and drinking behaviors, independently of the effects of psychoactive compounds. Additionally, we also verified that the associations observed are not due to the genetic overlap across the GSCAN phenotypes or to other genetically correlated psychiatric and behavioral traits including psychopathology, risk tolerance, educational attainment, and socioeconomic status.

## Materials and methods

### Philadelphia Neurodevelopmental Cohort

Phenotype and genotype data for PNC participants were obtained, after authorized access, from the National Center for Biotechnology Information database of Genotypes and Phenotypes (dbGaP; available at http://www.ncbi.nlm.nih.gov/gap) through dbGaP accession number phs000607.v3.p2 (Neurodevelopmental Genomics: Trajectories of Complex Phenotypes). The PNC is a population-based sample including more than 9500 individuals aged 8–21 years not enriched for any epidemiologically ascertained specific disorder, behavior, or trait [21]. The PNC participants were selected after stratification by sex, age, and ethnicity from a pool of approximately 50,000 subjects previously recruited from patients undergoing bloodwork in the Children’s Hospital of Philadelphia care network [22, 23]. Each participant was assessed for psychiatric and behavioral traits with a structured interview and completed a CNB following the Kiddie-SADS Family Study Interview [24, 25]. The structured interview included a panel of questions related to demographics, the timeline of life events, education, medical history, psychopathological assessment, and a global assessment of cognitive and executive functioning. The screening for symptoms related to psychiatric diagnoses was based on items defined by the fourth edition of the Diagnostic and Statistical Manual of Mental Disorders (DSM-IV) [26]. The CNB consisted of 14 tests assessing executive control, episodic memory, complex cognition, social cognition, and sensorimotor speed. A complete list of neurodevelopmental domains assessed and the specific features of each domain tested in the neurocognitive battery is available at https://www.ncbi.nlm.nih.gov/projects/gap/cgi-bin/study.cgi?study_id=phs000607.v3.p2. In our analysis, we tested phenotypes that were assessed in at least 500 participants. Supplementary Table 1 reports the sample size for each phenotype investigated. These included a total of 433 traits (Supplementary Table S1) that were grouped in 18 domains: attention deficit disorder, depression, generalized anxiety disorder, neuropsychiatric assessment, mania/hypomania, medical concerns, obsessive-compulsive disorder, oppositional defiant disorder, panic disorder, specific phobia, psychosis, post-traumatic stress, general probes, separation anxiety, structured interview for prodromal symptoms (SIPS) to assess psychotic risk, social anxiety, substance use, and other (i.e., phenotypes not classifiable in previous domains).

To account for the overall health, we included medical rating assessed at the administration of the tests among the covariates of the regression models. Additionally, we included the type of interview as a further covariate in our model to account for possible differences among participants in the assessment. Indeed, as for participants 8–10 years of age, the assessment was not direct, but caregivers or legal guardians were asked for information regarding the subject tested, and for probands aged 11–17, both the participants and their caregivers/legal guardians were interviewed. For the latter group, we investigated only the self-reported information to avoid a duplicated assessment. We decided to not explore differences in the PRS association between data derived from participants’ interviews and from caregivers’ interviews because of the limited sample size of the probands aged 11–17. The inclusion of “medical rating” and “type of interview" variables as covariates is in line with the design of previous PNC studies [27–29].

Our analysis was restricted to PNC participants of genetically confirmed European descent due to the lack of availability of large-scale GWAS data for other populations and known biases of cross-ancestry PRS analysis [30]. Considering these inclusion criteria, we investigated 433 psychiatric and behavioral traits (Supplementary Table S1) in 4498 children and young adults of European descent. Data quality control was performed as detailed in Wendt et al. [31]. Briefly, preimputation quality control was performed using PGC analysis pipeline specifically designed to handle datasets consisting of multiple genotyping platforms (see https://sites.google.com/a/broadinstitute.org/ricopili/preimputation-qc). Individuals of European descent were verified with genetic information via principal component analysis and the 1000 Genomes Project reference panel for populations with European ancestry (*N* = 503). For sample pairs with relatedness PI-HAT > 0.2, the sample with more informative phenotypes was retained. Imputation was performed for unrelated individuals of European ancestry using SHAPEIT for pre-phasing, IMPUTE2 for imputation, and the human 1000 Genomes Project Phase 3 as a reference panel [32–34].

### Genome-wide association data

Genome-wide association data for drinking and smoking traits were derived from GSCAN [13] and accessed via the Data Repository of the University of Minnesota (available at https://conservancy.umn.edu/handle/11299/201564). GSCAN GWAS included only individuals of European descent. GWAS data were generated in each study included in the GSCAN GWAS using RVTEST [35], accounting for the family-based studies and unrelated samples [33]. GSCAN investigated five traits, one related to alcohol drinking and four related to tobacco smoking. DPW (*N* = 941,280) was defined based on the average number of alcoholic drinks a participant reported consuming in a week. SI (*N* = 1,232,091) is a binary trait considering regular smokers as cases and non-smokers as controls, while ASI (*N* = 341,427) is a quantitative trait related to the age when an individual started to smoke tobacco-based cigarettes. SC (*N* = 547,219) was defined considering current smokers as cases and former smokers as controls. CPD (*N* = 337,334) was calculated in both current and former smokers by binning the quantitative measure of CPD (bin1 = 1–5; bin2 = 6–15; bin3 = 16–25; bin4 = 26–35; bin5 = 36+). Considering the risk variants identified in the GSCAN GWAS and available in the PNC cohort, we observed several associations (*p* < 0.05) with substance use phenotypes (Supplementary Table S2) although only 21% of the participants are informative for these phenotypes. The limited sample size and the characteristics of the PNC cohorts do not permit us to investigate single-variant effects.

### SNP-based heritability and genetic correlation

SNP-based heritability and genetic correlation for the smoking and drinking traits, and the additional phenotypes were estimated using the Linkage Disequilibrium Score Regression (LDSC) method [36, 37]. As provided by the LDSC developers (details available at https://github.com/bulik/ldsc), the analysis was conducted considering the HapMap 3 reference panel and pre-computed LD scores based on the 1000 Genomes Project reference data for individuals of European ancestry.

### PRS analysis

PRS based on drinking and smoking GWAS data were investigated with respect to psychiatric and behavioral traits assessed in the PNC (Supplementary Table S1). PRSice v. 2.3.1.c [38] was used to compute PRS using the clumping-thresholding method where the clumping step is used to obtain independent effect estimates from base datasets (smoking and drinking GWAS in the present study) and the thresholding step is used to maximize the predictive ability of the derived polygenic scores [39]. SNPs were clumped based on 250 kb windows, based on clump-r2 threshold = 0.1 and clump-p threshold = 1, respectively. The step size of the threshold was set to 5e−05, and the range of *p* value thresholds was from 5e−08 to 1 using an additive model for regression at each threshold. Although epistatic models could provide additional information regarding the genetics of drinking and smoking behaviors [40], a recent study showed that additive variance should account for the vast majority of the SNP-based heritability of complex traits [41]. All PRSs were covaried for age, sex, the first ten within-ancestry principal components (PCs), medical rating (i.e., the PNC code indicating the severity of a patient’s medical condition), and type of interview. False discovery rate (FDR) at 5% was applied to correct the results obtained for multiple testing, accounting for the number of phenotypes and PRS tested. The Manhattan plot related to the phenome-wide association study using the phenotypic binning was generated in R using the ggplot2 package [42]. To assess the independence of the phenotypes identified as associated with drinking and smoking PRS, we also calculated Spearman’s rank-order correlations via R using the rcorr function of the Hmisc library [43]. Correlation *p* values were adjusted for the number of tests performed using FDR *q* < 0.05. Finally, we verified whether the significant PRS associations with psychiatric and behavioral traits were independent of the genetic correlation among alcohol and tobacco phenotypes and between them and other psychiatric and behavioral traits. Results that survived multiple testing correction in the initial analysis were subsequently tested in two additional models. In model 2, we included as covariates the other PRS related to alcohol and tobacco use. Accordingly, if two among PRS tested are associated with the same trait and the association remained significant after covarying for each other effect, we can assume that the effects observed are independent of each other.

To verify whether the PRS associations were due to the genetic overlap of alcohol drinking and tobacco smoking with other complex traits, significant PRS associations were also covaried using PRS related to other psychiatric and behavioral traits in addition to the covariates defined in the model 1. Specifically, we used large-scale GWAS datasets, including Psychiatric Genomics Consortium cross-disorder (PGC-CD; *N* = 438,997 [44]), Social Science Genetic Association Consortium (SSGAC) cognitive performance (*N* = 257,828 [45]), SSGAC educational attainment (*N* = 766,345 [45]), SSGAC general-risk-tolerance (*N* = 466,571 [46]), and household income (*N* = 286,301 [47]). The PGC-CD study is a cross-disorder analysis including anorexia nervosa, attention-deficit/hyperactivity disorder, autism spectrum disorder, bipolar disorder, major depression, obsessive-compulsive disorder, schizophrenia, and Tourette syndrome [44]. We used PGC-CD GWAS data to account for the genetic overlap of alcohol and tobacco use with psychopathology and psychiatric comorbidities. SSGAC educational attainment (EA) was defined by mapping the major educational qualification of each participant to relevant categories from the International Standard Classification of Education (ISCED), then imputing the years-of-education equivalent for each ISCED category, facilitating comparison across different systems [45]. The SSGAC cognitive performance (CP) data were generated by meta-analyzing data from the COGENT (Cognitive Genomics Consortium) study and the UK Biobank (UKB). The COGENT study analyzed a phenotype defined as the first principal component derived from three or more neuropsychological tests [46]. In UKB, cognitive performance was defined based on the respondent’s score on a test of verbal-numerical reasoning. The SSGAC GWAS of general-risk tolerance (GR) meta-analyzed cohorts with different assessments capturing an individual’s tendency, preparedness, or willingness to take risks in general [47]. Annual household income (HI) GWAS was conducted in UKB using self-reported pre-tax household income binned to create a five-point scale (bin1 < £18,000, bin2 = £18,000–£29,999, bin3 = £30,000–£51,999, bin4 = £52,000–£100,000, bin5 > £100,000) [48]. HI data were analyzed as a proxy of socioeconomic status.

### Enrichment analysis

The SNPs used to generate each significant PRS association were analyzed for pathway enrichment using PRSet implemented in PRSice v. 2.3.1.c [38, 49]. Briefly, the PRSset method stratifies the PRS by gene sets. In our study, we used the Molecular Signatures Database (MSigDB) to derive gene sets related to gene ontologies and defined gene boundaries using the human gene annotation (GTF file). REVIGO [50] was employed to summarize GO terms by removing redundant items based on Jiang and Conrath semantic distance [51] and a similarity degree of 0.5.

## Results

### SNP-based heritability and genetic correlation

**Fig. 1:**
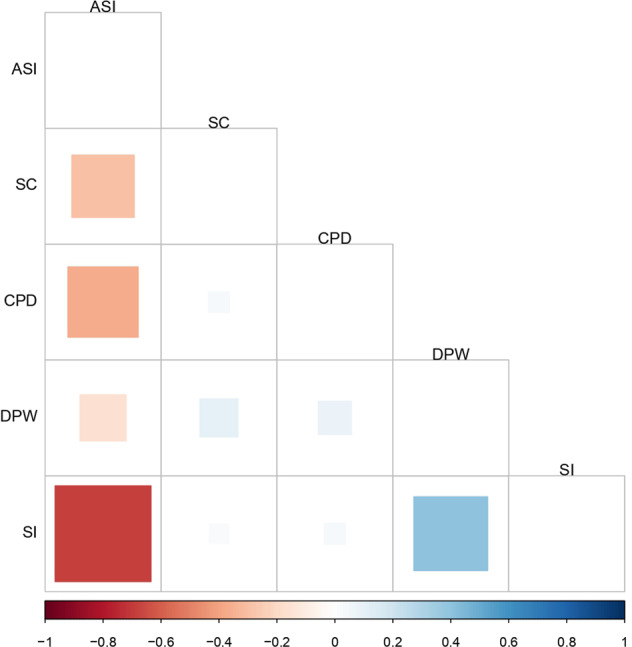
Genetic correlation matrix among smoking and drinking traits assessed by the GWAS & Sequencing Consortium of Alcohol and Nicotine. The square size is proportional to the magnitude of the correlation. Blank squares relate to not significant correlations (*P* > 0.01). ASI age of smoking initiation, CPD cigarettes per day, DPW drinks per week, SC smoking cessation, SI smoking initiation.

The SNP-based heritability of the alcohol-drinking and tobacco-smoking traits ranged from 0.032 ± 0.002 for SC to 0.072 ± 0.007 for CPD (Supplementary Table S3). LDSC-based correlations were examined among the substance-use phenotypes. Their genetic correlations are highly significant, but their absolute value ranges from 0.083 to 0.684. DPW showed a positive genetic correlation with each tobacco-smoking trait (SI rg = 0.407, *P* = 1.40E−92; CPD rg = 0.083, *P* = 3.93E−03; SC rg = 0.108, *P* = 1.02E−03; Fig. 1 and Supplementary Table S4) with the exception of ASI (rg = −0.160, *P* = 2.73E−07). Similarly, ASI is negatively genetically correlated with the other tobacco-smoking phenotypes (ASI vs. SI, rg = −0.684, *P* = 3.09E−199; ASI vs. CPD, rg = −0.369, *P* = 1.07E−23; ASI vs. SC, rg = −0.291, *P* = 1.71E−12). Considering other psychiatric, behavioral, and social traits (Supplementary Table S5), PGC-CD showed a positive genetic correlation with CPD (rg = 0.21, *P* = 2.13E−18), SI (rg = 0.215, *P* = 2.33E−26), DPW (rg = 0.107, *P* = 6.01E−06), and SC (rg = 0.102, *P* = 1.54E−03), while a negative correlation was present with respect to ASI (rg = −0.147; *P* = 9.22E−09). CP was positively correlated with ASI (rg = 0.314, *P* = 6.31E−32), while the other smoking traits showed only a weak negative rg (CP vs. SI rg = −0.172, *P* = 2.34E−22; CP vs. CPD rg = −0.103, *P* = 2.35E−05, and CP vs. SC rg = −0.298, *P* = 2.80E−28). EA was the most correlated additional behavioral trait respect to ASI (rg = 0.599, *P* = 7.08E−167), SC (rg = −0.502, *P* = 4.15E−95), SI (rg = −0.362, *P* = 2.75E−128), and CPD (rg = −0.285, *P* = 8.32E−29). HI followed the same correlation pattern of EA, while GR is the only adjunctive trait negatively related to ASI (rg = −0.228, *P* = 8.46E−13), showing a positive association with the other smoking phenotypes (GR vs. SI rg = 0.327, *P* = 1.54E−46; R vs. CPD rg = 0.175, *P* = 7.87E−07; GR vs. SC rg = 0.180, *P* = 4.22E−07). Finally, GR was also the only additional trait with a moderate positive genetic correlation with DPW (rg = 0.286, *P* = 4.26E−30). Supplementary Table S4 provides details of the genetic correlations calculated among the GWAS datasets investigated.

### Phenotypic correlations among psychiatric and behavioral traits associated with smoking and drinking polygenic risk

**Fig. 2:**
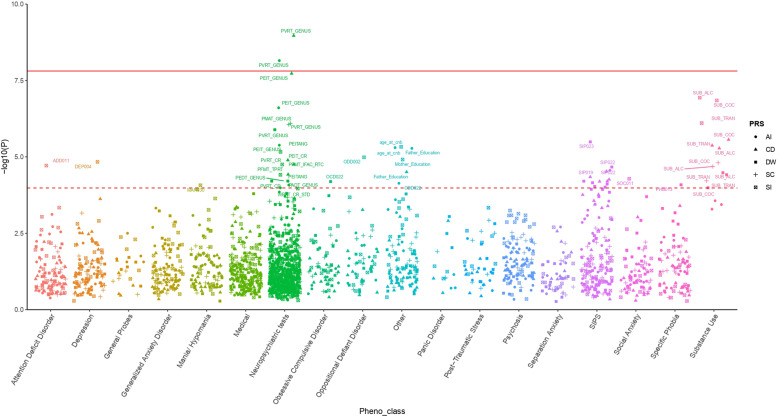
Association of drinking and smoking polygenic risk scores for 433 psychiatric and behavioral traits. The dashed line represents the FDR 5% threshold, while the solid line refers to Bonferroni 5% correction. The definition of the phenotype abbreviations is available in Supplementary Table S1. ASI age of smoking initiation, CPD cigarettes per day, DPW drinks per week, SC smoking cessation, SI smoking initiation.

**Fig. 3:**
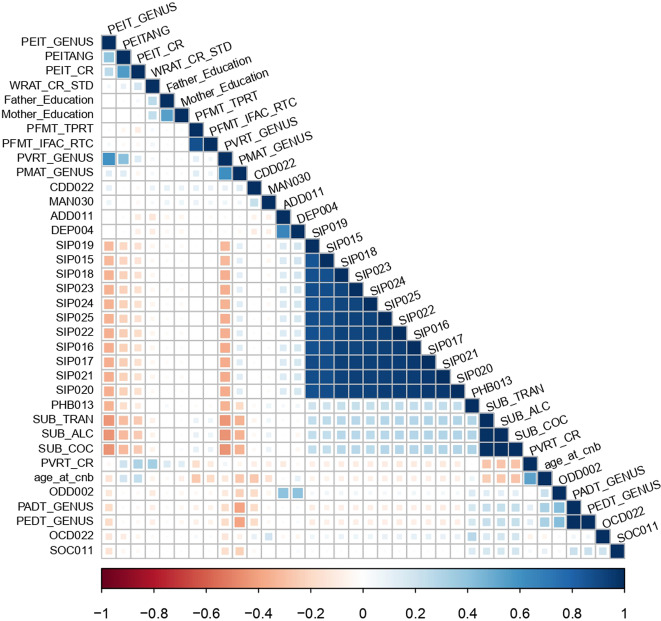
Spearman’s rank-order correlation matrix across 36 neurobehavioral traits significantly associated with the PRS analyzed. The square size is proportional to the magnitude of the correlation. Blank squares relate to not significant correlations (*P* > 0.01). The definition of the phenotype abbreviations is available in Supplementary Table S1.

The polygenic risk drinking and smoking behaviors were tested with respect to 433 psychiatric and behavioral traits assessed in PNC children and young adults and a total of 36 phenotypes were significantly associated after accounting for the number of PRS and phenotypes tested (FDR < 5%; Table 1, Fig. 2 and Supplementary Table S6). To assess the independence of the phenotypes identified as associated with drinking and smoking PRS, we conducted a Spearman’s correlation analysis among these psychiatric and behavioral traits, observing several significant correlations (Fig. 3 and Supplementary Table S7). We observed a high correlation among the psychosis-related SIPS outcomes [52] (Spearman’s *ρ* > 0.87, *P* < 0.001). Penn computerized individual tests outcomes were also positively correlated to each other: Median Response Time for Correct Responses to Target Faces (PFMT_TPRT) and Median Response Time for Total Correct Test Trial Responses (PFMT_IFAC_RTC) (Spearman’s *ρ* = 0.87, *P* < 0.0001) for Penn Face Memory Test; Penn Verbal Reasoning Test Genus (PVRT_GENUS) and Penn Matrix Reasoning Test Genus (PMAT_GENUS) (Spearman’s *ρ* = 0.61, *P* < 0.0001) for verbal and reasoning tests; Penn Age Differentiation Test (PADT_GENUS) and Penn Emotion Differentiation Test (PEDT_GENUS) (Spearman’s *ρ* = 1, *P* < 0.0001) for age and emotion recognition. Alcohol, cocaine, and tranquilizer use were also highly correlated (Spearman’s *ρ* > 0.98, *P* < 0.0001).

**Table 1:** Association of smoking and drinking PRS with psychiatric and behavioral traits surviving the FDR 5% threshold.

PRS	Phenotype	Threshold	SNP *N*	Model 1			Model 2			Model 3		
				*R*^2^	*Z-* score	*P*	*R*^2^	*Z-* score	*P*	*R*^2^	*Z-* score	*P*
ASI	PEITANG	0.408	59,052	0.49%	4.61	4.14E−06	0.44%	4.36	1.31E−05	0.21%	3.06	0.002
	Father_Education	0.0016	897	0.47%	4.57	5.00E−06	0.41%	4.27	2.03E−05	0.28%	3.62	3.04E−04
	SIP019	0.1	21,081	0.41%	−4.2	2.73E−05	0.34%	−3.84	1.24E−04	0.15%	−2.52	0.012
	CDD022	0.093	19,832	0.34%	3.97	7.34E−05	0.28%	3.62	3.03E−04	0.25%	3.4	6.76E−04
	WRAT_CR_STD	1	99,097	0.35%	3.89	1.03E−04	0.28%	3.46	5.36E−04	0.05%	1.47	0.142
CPD	PVRT_GENUS	0.0013	1039	0.82%	−6.11	1.08E−09	0.72%	−5.8	7.20E−09	0.41%	−4.44	9.35E−06
	PEIT_GENUS	4.00E−04	520	0.69%	−5.63	1.87E−08	0.49%	−4.78	1.83E−06	0.33%	−3.99	6.76E−05
	PEIT_CR	2.50E−04	413	0.42%	−4.37	1.28E−05	0.35%	−3.96	7.51E−05	0.28%	−3.57	3.66E−04
	PVRT_CR	0.0015	1159	0.31%	−4.3	1.77E−05	0.31%	−4.26	2.07E−05	0.15%	−3.09	0.002
	SIP017	0.0013	1039	0.39%	4.08	4.54E−05	0.33%	3.82	1.37E−04	0.20%	2.95	0.003
	SIP018	0.0013	1039	0.38%	4.04	5.48E−05	0.27%	3.42	6.24E−04	0.16%	2.64	0.008
	SIP020	0.0013	1039	0.37%	4	6.37E−05	0.35%	3.92	8.82E−05	0.20%	2.99	0.003
DPW	SIP023	0.0024	2025	0.50%	−4.66	3.23E−06	0.44%	−4.37	1.28E−05	0.36%	−4	6.37E−05
	SIP022	0.0059	3468	0.42%	−4.25	2.15E−05	0.35%	−3.89	1.03E−04	0.27%	−3.42	6.24E−04
	PADT_GENUS	5.00E−08	48	0.31%	4.01	6.18E−05	0.28%	3.81	1.43E−04	0.28%	3.82	1.33E−04
	PEDT_GENUS	5.00E−08	48	0.31%	4.01	6.18E−05	0.28%	3.81	1.43E−04	0.28%	3.82	1.33E−04
	SIP021	0.0059	3468	0.37%	−4.01	6.24E−05	0.30%	−3.59	3.30E−04	0.22%	−3.12	0.002
	OCD022	5.01E−05	299	0.38%	4	6.42E−05	0.38%	4.01	6.18E−05	0.34%	3.8	1.44E−04
	SIP015	0.0071	3877	0.37%	−3.98	7.03E−05	0.30%	−3.58	3.42E−04	0.24%	−3.22	0.001
	SIP016	0.0071	3893	0.36%	−3.96	7.76E−05	0.38%	−4.06	4.92E−05	0.28%	−3.48	4.99E−04
	PHB013	5.01E−05	299	0.36%	3.94	8.19E−05	0.39%	4.1	4.26E−05	0.32%	3.72	2.02E−04
	SIP024	0.0071	3893	0.36%	−3.91	9.27E−05	0.39%	−4.12	3.87E−05	0.30%	−3.62	2.99E−04
	SIP025	0.0024	2025	0.35%	−3.9	9.79E−05	0.32%	−3.73	1.92E−04	0.27%	−3.47	5.27E−04
SC	PMAT_GENUS	5.01E−05	73	0.51%	4.94	8.06E−07	0.50%	4.91	9.23E−07	0.46%	4.72	2.47E−06
SI	SUB_ALC	0.077	20,981	0.58%	−5.31	1.16E−07	0.68%	−5.78	7.83E−09	0.90%	−6.72	2.04E−11
	SUB_COC	0.072	20,123	0.57%	−5.27	1.42E−07	0.66%	−5.72	1.17E−08	0.89%	−6.69	2.58E−11
	SUB_TRAN	0.077	20,981	0.51%	−4.95	7.85E−07	0.61%	−5.44	5.76E−08	0.83%	−6.4	1.67E−10
	age_at_cnb	0.072	20,138	0.01%	4.58	4.70E−06	0.02%	5.16	2.56E−07	0.02%	5.6	2.35E−08
	ODD002	0.149	32,093	0.69%	4.41	1.03E−05	0.74%	4.58	4.65E−06	0.40%	3.37	7.64E−04
	Mother_Education	0.081	21,799	0.44%	−4.38	1.22E−05	0.36%	−3.95	7.88E−05	0.25%	−3.35	8.28E−04
	DEP004	0.01	6639	0.63%	4.34	1.45E−05	0.57%	4.11	4.04E−05	0.43%	3.58	3.42E−04
	PFMT_IFAC_RTC	0.012	7229	0.42%	−4.3	1.77E−05	0.36%	−4	6.42E−05	0.25%	−3.33	8.62E−04
	ADD011	5.01E-05	677	0.59%	4.27	1.93E−05	0.45%	3.74	1.87E−04	0.31%	3.13	0.002
	PFMT_TPRT	0.011	6963	0.37%	−4.12	3.86E−05	0.39%	−4.21	2.56E−05	0.25%	−3.42	6.35E−04
	SOC011	0.0084	5875	0.38%	−4.05	5.21E−05	0.36%	−3.94	8.45E−05	0.27%	−3.41	6.60E−04
	MAN030	0.0078	5636	0.36%	−3.93	8.48E−05	0.34%	−3.83	1.30E−04	0.23%	−3.15	0.002

The definition of the phenotype abbreviations is available in Supplementary Table S1. The results were assessed with respect to three regression models, including different sets of covariates. Model 1 covariates: 10 principal components, Sex, Age, Type of Interview, Medical Rating; Model 2 covariates: Model 1 + PRSs for smoking and alcohol traits; Model 3 covariates: Model 1 + PRSs for PGC cross-disorder, cognitive performance, educational attainment, general-risk tolerance, and household income.

*ASI* age of smoking initiation, *CPD* cigarettes per day, *DPW* drinks per week, *SC* smoking cessation, *SI* smoking initiation.

### Drinks per week

The PRS for DPW was negatively associated with seven outcomes derived from the psychosis-related SIPS (SIP015: “I think I have felt that there are odd or unusual things going on that I can’t explain” *Z*-score = −3.98, *R*^2^ = 0.4%, *P* = 7.03E−05; SIP016: “I think that I might be able to predict the future” *Z*-score = −3.96, *R*^2^ = 0.4%, *P* = 7.76E−05; SIP021: “I wonder if people may be planning to hurt me or even may be about to hurt me” *Z*-score = −4.01, *R*^2^ = 0.4%, *P* = 6.24E−05; SIP022: “I believe that I have special natural or supernatural gifts beyond my talents and natural strengths” *Z*-score = −4.25, *R*^2^ = 0.4%, *P* = 2.15E−05; SIP023: “I think I might feel like my mind is “playing tricks” on me” *Z*-score = −4.66, *R*^2^ = 0.5%, *P* = 3.23E−06; SIP024: “I have had the experience of hearing faint or clear sounds of people or a person mumbling or talking when there is no one near me” *Z*-score = −3.91, *R*^2^ = 0.4%, *P* = 9.27E−05; SIP025: “I think that I may hear my own thoughts being said out loud” *Z*-score = −3.90, *R*^2^ = 0.4%, *P* = 9.79E−05). DPW polygenic risk was positively associated with the specific phobia-related item (PHB013: “Thinking about all of the time that you were afraid of (insert worst fear), whether or not you actually faced it, how long did this fear last? (Weeks)” *Z*-score = 3.94, *R*^2^ = 0.4%, *P* = 8.19E−05), one of the obsessive-compulsive disorder traits (OCD022 *Z*-score = 4.00, *R*^2^ = 0.4%, *P* = 6.42E−05), and PADT_GENUS and PEDT_GENUS (Penn Age Differentiation and Emotion Differentiation Test, both featuring a *Z*-score = 4.01, *R*^2^ = 0.3%, *P* = 6.18E−05).

### Age of smoking initiation

The PRS for ASI was positively associated with the ability to recognize the angry facial emotions of others (PEITANG: *Number of Correct Responses to Anger Trials*, *Z*-score = 4.61, *R*^2^ = 0.5%, *P* = 4.14E−06), the standardized score from the Wide Range Achievement Test [53] (WRAT_CR_STD: *age-adjusted WRAT test determining participants’ ability to complete the battery and to provide an estimate of IQ*, *Z*-score = 3.89, *R*^2^ = 0.3%, *P* = 1.03E–04); an item related to conduct disorder (CDD022: “How much did these behaviors change your relationships with your friends?” *Z*-score = 3.97, *R*^2^ = 0.3%, *P* = 7.34E–05), and years of father’s education (*Z*-score = 4.57, *R*^2^ = 0.5%, *P* = 5.00E-06). The only negative association was found with respect to item SIP019 (“I think that I may get confused at times whether something I experience or perceive may be real or may be just part of my imagination or dreams” *Z*-score = −4.20, *R*^2^ = 0.4%, *P* = 2.73E−05).

### Smoking initiation

The polygenic risk of SI positively associated with the age at completion of the CNB (*Z*-score = 4.58, *R*^2^ = 0.01%, *P* = 4.70E−06), one of the oppositional defiant disorder traits (ODD002: “Was there a time when you often got into trouble with adults for refusing to do what they told you to do or for breaking rules at home/school” *Z*-score = 4.41, *R*^2^ = 0.7%, *P* = 1.03E−05), a depression-related item (DEP004: “Has there ever been a time when you felt grouchy, irritable or in a bad mood most of the time; even little things would make you mad?” *Z-*score = 4.34, *R*^2^ = 0.6%, *P* = 1.45E−05), and attention deficit disorder (ADD011: “Did you often have trouble paying attention or keeping your mind on your school, work, chores, or other activities that you were doing?” *Z*-score = 4.27, *R*^2^ = 0.6%, *P* = 1.93E−05). A negative association was observed for the PRS of SI and the years of mother’s education (*Z*-score = −4.38, *R*^2^ = 0.4%, *P* = 1.22E−05), social anxiety (SOC011: “Thinking about all of the time that you were afraid of (insert worst fear), whether or not you actually faced it, how long did your fear of this situation last? (Months)” *Z*-score = −4.05, *R*^2^ = 0.4%, *P* = 5.21E−05), and a mania-related item (MAN030: “How much did your feeling (too happy/excited/grouchy/energetic) upset or bother you?” *Z*-score = −3.93, *R*^2^ = 0.4%, *P* = 8.48E−05). The two highly correlated phenotypes accounting for the Penn Face Memory Test were also negatively associated with the PRS of SI (PFMT_TPRT: *Median Response Time for Correct Responses to Target Faces* *Z*-score = −4.12, *R*^2^ = 0.4%, *P* = 3.86E−05, and PFMT_IFAC_RTC: *Median Response Time for Total Correct Test Trial Responses* *Z*-score = −4.30, *R*^2^ = 0.4%, *P* = 1.77E−05, respectively). SI polygenic risk was also negatively associated with the alcohol (*Z*-score = −5.31, *R*^2^ = 0.6%, *P* = 1.16E−07), cocaine (*Z*-score = −5.27, *R*^2^ = 0.6%, *P* = 1.42E−07), and tranquilizer (*Z*-score = −4.95, *R*^2^ = 0.5%, *P* = 7.85E−07) endorsement phenotypes.

### Cigarettes per day

The PRS for CPD was positively associated with three SIPS-derived psychosis outcomes (SIP017: “I may have felt that there could possibly be something interrupting or controlling my thoughts, feelings, or actions” *Z*-score = 4.08, *R*^2^ = 0.4%, *P* = 4.54E−05, SIP018: “I have had the experience of doing something differently because of my superstitions” *Z*-score = 4.04, *R*^2^ = 0.4%, *P* = 5.48E−05, and SIP020: “I have thought that it might be possible that other people can read my mind, or that I can read others’ minds” *Z*-score = 4.00, *R*^2^ = 0.4%, *P* = 6.37E−05. Furthermore, the Penn Emotion Identification Test-related traits and those included in the CNB for the Penn Verbal Reasoning Test were negatively associated to PRS for CPD (PEIT_GENUS *Z*-score = −5.64, *R*^2^ = 0.7%, *P* = 1.87E−08, and PEIT_CR *Z*-score = −4.37, *R*^2^ = 0.4%, P = 1.28E−05, PVRT_GENUS *Z*-score = −6.11, *R*^2^ = 0.8%, *P* = 1.08E−09, and PVRT_CR *Z*-score = −4.30, *R*^2^ = 0.3%, *P* = 1.77E−05).

### Smoking cessation

After multiple testing correction, a single association was found for SC polygenic risk with respect to the ability to perform the Penn Matrix Reasoning Test, which assesses reasoning by geometric analogy and contrast principle (PMAT_GENUS *Z*-score = 4.94, *R*^2^ = 0.5%, *P* = 8.06E−07).

### Independence of PRS associations with respect to the genetic overlap among alcohol drinking, tobacco smoking, psychopathology, and other behavioral traits

**Fig. 4:**
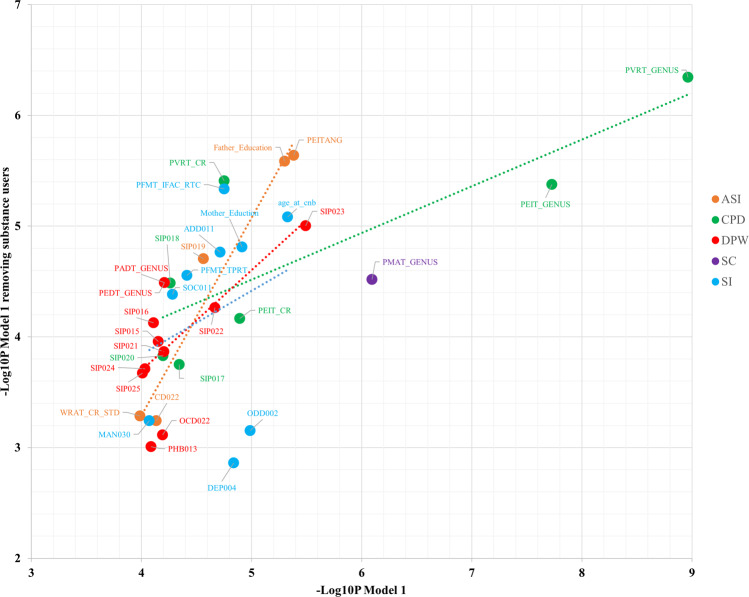
Relationship between the statistical significance (−log_10_*P*value) of PRS association including and excluding participants endorsing substance use (*x* axis and *y* axis, respectively). The analyses were conducted on the covariates of model 1 (i.e., ten principal components, Sex, Age, Type of Interview, Medical Rating). The definition of the phenotype abbreviations is available in Supplementary Table S1. The dashed lines represent the linear relationship between the results obtained from these analyses with respect to the different drinking and smoking polygenic risk scores tested. PRS polygenic risk score, DPW drinks per week, ASI age of smoking initiation, SI smoking initiation, CPD cigarettes per day, SC smoking cessation, PGC-CD Psychiatric Genomics Consortium Cross Disorder, CP cognitive performance, EA educational attainment, GR general-risk-taking behavior, HI household income, PNC Philadelphia Neurodevelopmental Cohort.

To test whether the associations of drinking and smoking polygenic risk with psychiatric and behavioral traits were due to the genetic overlap with psychopathology, EA, CP, GR, and HI, we added additional covariates to the regression model used in the initial analysis (model 1). Building upon the sets of covariates included in model 1 (i.e., sex, age, the first ten within-ancestry PCs, medical rating, and type of interview), we added the PRS of the other alcohol-drinking and tobacco-smoking traits as covariates (model 2). In model 3, the PRS for psychopathology (i.e., PGC-CD), EA, CP, GR, and HI were added to the set of covariates included in model 1. The significant associations observed in model 1 remained significant in the model 2 and model 3 analysis with the exception of the association of PRS for ASI with WRAT_CR_STD (“age-adjusted WRAT test determining participants ability to complete the battery and to provide an estimate of IQ”) when model 3 covariates were applied (model 1: *Z*-score = 3.89, *R*^2^ = 0.35%, *P* = 1.03E−04; model 3: *Z*-score = 1.47, *R*^2^ = 0.05%, *P* = 0.142). To further test the independence of the PRS associations from the effects of psychoactive substance use, we removed the participants endorsing the use of alcohol or drugs (*N* = 964; 21%). Applying model 1 covariates, we observed consistent PRS associations between the full PNC cohort and the sample excluding substance users (Fig. 4 and Supplementary Table S8).

### Enrichment analysis

We interrogated the biological processes to characterize the molecular mechanisms underlying the alcohol-drinking and tobacco-smoking PRS associations, identifying several GO enrichments surviving Bonferroni multiple testing correction (Supplementary Table S9). The associations identified for CPD polygenic risk involved several biological domains, including *neuromuscular junction development* (GO:0007528, *P* = 3.50E−15) and *amyloid precursor protein metabolic process* (GO:0042982, *P* = 1.68E−13), among the most significant. Strong enrichments related to immune systems functions were also observed, including *leukocyte activation involved in inflammatory response* (GO:0002269, *P* = 1.62E−13), *positive regulation of interleukin-6 biosynthetic process* (GO:0045410, *P* = 2E−13), and *erythrocyte maturation* (GO:0043249, *P* = 2.61E−13). The genes underpinning the associations related to the ASI polygenic risk were enriched for several biological pathways, whose primary roles seemed to be linked to the muscle tissue and brain development, including *myotube cell development* (GO:0014904, *P* = 4.00E−8), *striated muscle cell differentiation* (GO:0051146, *P* = 5.53E−8), and *response to manganese ion* (GO:0010042, *P* = 7.17E−8). The associations of the PRS for DPW were enriched for *negative regulation of cell projection organization* (GO:0031345, *P* = 5.60E−8) and *negative regulation of cell development* (GO:0031345, *P* = 6.54E−8). With respect to the associations identified for the PRS for SI, we observed enrichment for *DNA geometric change* (GO:0032392, *P* = 2.34E−13), *cellular response to carbohydrate stimulus* (GO:0071322, *P* = 5.13E−12), *axodendritic protein transport* (GO:0099640, *P* = 1.75E−10), *positive regulation of Phospholipase A2 activity* (GO:0032430, *P* = 2.67E−10), *phosphatidylethanolamine acyl-chain remodeling* (GO:0036152, *P* = 1.05E−10), and *intracellular receptor signaling pathway* (GO:0030522, *P* = 1.13E−10). Finally, no enrichment related to the SC polygenic risk associations survived multiple testing correction.

## Discussion

Leveraging well-powered genome-wide information generated by the GSCAN study of alcoholic drinks-per-week and four traits related to tobacco smoking [13], we investigated the genetic liability for these five traits with respect to psychiatric and behavioral traits in children and young adults. The present study expands the findings provided by recent investigations of drinking and smoking PRS in predicting alcohol use disorder remission in adults and in dissecting the association of prenatal alcohol exposure and offspring alcohol use in mother-child pairs [54, 55]. Our findings increase the understanding of the possible psychiatric and behavioral consequences of smoking and alcohol polygenic risk in childhood and early adulthood. In particular, we observed a wide range of PRS associations and the majority of them were not affected by the genetic overlap with the psychopathology spectrum (i.e., PGC-CD), GR, EA, and HI, or to substance use among PNC participants. The strength of the PRS associations identified is in line with other cross-phenotype PRS analyses done with respect to psychiatric and behavioral traits [56–58]. Since the associations observed appear to be largely mutually independent, we discussed the results for each smoking/drinking PRS separately.

### Drinks per week

This was the only alcohol-related trait analyzed. The PRS was negatively associated with the outcome of several psychosis-related SIPS items capturing the time-length of the specific symptoms as part of an assessment of psychosis presence and severity [59]. Differently from the previously hypothesized “self-medication” and diathesis-stress model [60], our results indicated that children and young adults with low genetic liability to alcohol drinking reported longer periods of prodromal psychotic symptoms. This is the opposite of what is expected for problematic alcohol use and alcohol dependence. Accordingly, we hypothesize that the association in our study is due to the “moderate alcohol consumption” component present in DPW and not to the component of this trait associated with alcohol misuse, reflecting the genetic differences between DPW and alcohol use disorder [61].

The main biological processes underlying the PRS associations correspond to the negative regulation of critical cell activities such as the development and the projection organization, ultimately impacting brain activity. Aberrations in these processes have previously been implicated in neurodevelopmental changes underlying psychosis [62, 63] and in altered synapses in the limbic brain areas that drive drinking behavior [64, 65]. DPW polygenic risk was also associated with the number of test trials administered to evaluate the social cognition and behavioral function in the psychometric tests related to emotion differentiation and age differentiation [66–68]. Finally, PRS for DPW was also positively associated with anxiety-related obsessive-compulsive disorder. The mesolimbic dopaminergic pathway originating in the ventral tegmental area (VTA) is critical for the onset of reward processing and emotional responses related to anxiety-related disorders [69–71].

### Age of smoking initiation

Individuals with high genetic liability to early smoking initiation had a longer duration of psychosis symptoms. Despite the well-known association between schizophrenia, and cigarette smoking, a previous study suggested that individuals with psychosis started smoking at a similar age as non-psychotic comparison subjects [72]. Our findings are consistent with the hypothesis that psychosis and the predisposition to early smoking share some genetic liability. PRS for ASI is, unsurprisingly, positively associated with the years of paternal education. Adolescents whose parents had less or no college education are much more likely to smoke and to smoke earlier than those whose parents have a higher education [73]. Our results showed that the association between ASI polygenic risk and father’s education held even when the polygenic components of educational attainment, cognitive performance, and socioeconomic status were added as covariates in the model. This suggests that other mechanisms (e.g., dynastic effects and assortative mating) may be responsible for the genetic overlap between tobacco-smoking behaviors and parents’ education.

ASI genetic liability was positively associated with higher scores for correct recognition of angry faces (PEITANG) during the Penn Emotional Identification Test. This relationship suggests that genetic predisposition for smoking initiation may share some liability toward preferential processing of negative social information or perhaps a heightened experience of interpersonal stressors [74, 75]. This association was enriched for biological processes mainly related to cellular response to manganese ions (Mn^2+^), which is a key element in brain activation induced by chronic psychosocial stress [76, 77].

The genetic predisposition to later smoking initiation was also positively associated with the ratings of the effects of conduct disorder impacting social relationships (i.e., referencing a pattern of disruptive and violent behaviors following rule-breaking encounters; CDD022: “How much did these behaviors change your relationships with your friends?”) suggesting a more consciousness of their disruptive and antisocial behavior in people starting smoking later. A shared genetic etiology among substance abuse and conduct disorder has been previously described [78], and our data extend this relationship to the age of smoking initiation.

The WRAT total standardized score (WRAT_CR_STD) [79] was positively associated with ASI polygenic risk. An impact of smoking on cognitive decline has been observed in adulthood [80, 81] and childhood [82]. Nonetheless, our study suggests that this relationship might be also due to a shared genetic predisposition rather than the sole effect of tobacco smoking. Indeed, this association is not significant when covaried for the other PRS investigated.

While early tobacco use was already associated with specific non-affective psychosis [83], the association of PRS for ASI with SIP019 phenotype (i.e., “I think that I may get confused at times whether something I experience or perceive may be real or maybe just part of my imagination or dreams”) supports a partial contribution from horizontal pleiotropy (i.e., shared genetic basis) between these traits.

### Cigarettes per day

PRS for CPD was positively associated with several psychosis-related SIPS items indicating the duration of prodromal psychotic symptoms. This finding is consistent with the shared genetic liability observed in discordant twin and sibling studies of schizophrenia [84]. With respect to this PRS association, we observed multiple biological processes related to cellular signaling homeostasis, including terms related to *Synaptic Vesicles Membrane* (GO:0030672) and *Signal Release* (GO:0023061). The disruption of synaptic plasticity is known to be associated with psychotic behaviors [85, 86].

The genetic liability to smoking quantity was negatively associated with emotional identification and verbal reasoning independently from psychopathology, substance use, and other behavioral traits. Accordingly, we can hypothesize that certain molecular mechanisms involved in the predisposition to smoking quantity are shared with these psychiatric and behavioral traits.

We observed multiple neuroinflammatory pathways among the biological processes enriched for CPD PRS associations. Neuroinflammation appears to correlate with neurocognitive changes in the context of aging [87–90]. Experimental manipulations of IL-6 signaling appear capable of producing related effects in animal models [91, 92]. Interaction between smoking history and genetic variation in the IL-6 promoter was predictive of circulating IL-6 and CRP levels [93]. Thus, pleiotropic effects between smoking quantity and cognitive development may involve the IL-6 pathway. Moreover, CPD genetic liability was previously shown to be enriched with biological processes related to amyloid metabolism and neuroinflammation [94].

### Smoking initiation and cessation

Genetic predisposition to smoking initiation was positively associated with items related to oppositional defiant disorder in children and young adults. The positive phenotypic correlation of ODD002 (Oppositional Defiant Disorder: *Was there a time when you often got into trouble with adults for refusing to do what they told you to do or for breaking rules at home/school?*) with ADD011 (Attention Deficit Disorder: *Did you often have trouble paying attention or keeping your mind on your school, work, chores, or other activities that you were doing?*) and DEP004 (Depression: *Has there ever been a time when you felt grouchy, irritable or in a bad mood most of the time; even little things would make you mad?*) was already described [95, 96] and might account for the associations’ consistency with SI genetic liability.

The dysregulations of dopaminergic and nicotinic pathways are shared mechanisms between smoking habits and the onset of attention deficit disorders [97]. Conversely, the relationship between tobacco smoking and depressive status and anxiety is inconsistent in terms of the direction of association [98]. Our results supported the sharing of genetic determinants for smoking initiation and these survey item reports, even when the genetic predisposition for psychopathology was included as a covariate in the model. Nevertheless, environmental factors, such as the family context and parental behaviors, play a crucial role in the onset of these behaviors [99–101]. Indeed, higher levels of maternal education were associated with a lower likelihood of being a smoker [4]. This association held on even after we included PRSs for psychosocial and psychopathological traits as covariates, supporting the interplay between familiar factors and SI genetic predisposition in early adolescence [102].

Two more traits, SOC011 (Social Anxiety: *Thinking about all of the time that you were afraid of your worst fear, whether or not you actually faced it, how long did your fear of this situation last in months?)* and the age when the computerized neurocognitive battery was completed, were associated with the SI genetic predisposition, and, as expected, it is also positively related to the substance use. A child with a higher genetic vulnerability for smoking initiation may have more behavioral issues in general [103], which might prompt parents to seek evaluation at an earlier age. Indeed, even though the PNC cohort is not enriched for any disorder, the participants were recruited through a pediatric healthcare network. Accordingly, PNC participants include individuals reporting psychiatric traits and outcomes. We hypothesize that the association of PRS for SI with age at CNB administration may reflect a participation bias in the PNC cohort.

The positive relationship between the PRS for SI and age at neurocognitive testing was enriched with genetic variants involved in brain-relevant biological pathways, including positive regulation of the phospholipase A2 (PLA2) (GO: 0032430), which is involved in a pro-inflammatory status [104]. It may also contribute to nervous system degeneration [105], and appears to impact iron accumulation in the globus pallidus, the substantia nigra, and the dentate nucleus [106]. Our data are consistent with the previously reported association between *PLA2* genetic variation and the development of smoking habits in people affected by psychiatric disorders [107]. Other genetic variants underpinning this association are also involved in the axodendritic protein transport (GO: 0099640), suggesting their role in the proper synapse development [108].

Our data confirm that tobacco smoking and alcohol drinking share a genetic liability involving multiple biological processes [109], and the most significant functional enriched process in substance use association was the DNA geometric change (GO:0032392).

Genetic variants underlying substance use associations with the genetic liability for tobacco smoking recognized cellular response to carbohydrate stimuli, that are increasingly considered to alter brain circuitry, leveraging the induction of dopamine reward and craving that are comparable in magnitude to those induced by addictive drugs or alcohol use [110, 111].

The substance associations with the genetic liability to SI were also driven by genetic variants involved in the metabolism of phosphatidylethanolamine, a phospholipid critical for white matter establishment [112, 113]. Finally, the genetic liability to SC partially overlaps with the outcome of the Penn Matrix Reasoning Test, suggesting that people who opted for fewer practice trials before the test are less genetically predisposed to quit smoking.

In conclusion, our study provided evidence that the polygenic risk for tobacco-smoking and alcohol-use phenotypes overlap with several neurobehavioral traits assessed in a population-based cohort of children and adolescence, and that these relationships appeared independent of actual psychoactive substance use. The associations were also independent of the genetic effects exerted by genetically correlated phenotypes, including other substance use phenotypes, psychopathology, and psychosocial factors. Our findings highlight plausible pleiotropic mechanisms linking genetic liability to smoking and drinking behaviors to aspects of cognitive and behavioral development.

## Supplementary information

Supplementary Table 1

Supplementary Table 2

Supplementary Table 3

Supplementary Table 4

Supplementary Table 5

Supplementary Table 6

Supplementary Table 7

Supplementary Table 8

Supplementary Table 9
