# Supplementary material for: Drinking and smoking polygenic risk is associated with childhood and early-adulthood psychiatric and behavioral traits independently of substance use and psychiatric genetic risk
Source: Transl Psychiatry. 2021 Nov 13;11:586. doi: 10.1038/s41398-021-01713-z (PMC8590689; doi:10.1038/s41398-021-01713-z)
Supplement: Supplementary file 3 — Supplementary Table 3 [file 41398_2021_1713_MOESM3_ESM.docx]

**Supplementary Table 3**: Estimates of SNP heritability of drinking and smoking behaviors based on the LD score regression method. ASI: Age of Smoking Initiation; DPW: Drinks per Week; SI: Smoking Initiation: CPD: Cigarettes per Day: SC: Smoking Cessation.
