# Supplementary material for: Drinking and smoking polygenic risk is associated with childhood and early-adulthood psychiatric and behavioral traits independently of substance use and psychiatric genetic risk
Source: Transl Psychiatry. 2021 Nov 13;11:586. doi: 10.1038/s41398-021-01713-z (PMC8590689; doi:10.1038/s41398-021-01713-z)
Supplement: Supplementary file 4 — Supplementary Table 4 [file 41398_2021_1713_MOESM4_ESM.docx]

**Supplementary Table 4**: Genetic correlations across the analyzed traits derived by LD score regression and GWAS summary statistics data available in the literature. The lower triangular matrix refers the genetic correlation (rg), and the upper triangular matrix reports the genetic correlation significance (P-values).
