# Supplementary material for: Drinking and smoking polygenic risk is associated with childhood and early-adulthood psychiatric and behavioral traits independently of substance use and psychiatric genetic risk
Source: Transl Psychiatry. 2021 Nov 13;11:586. doi: 10.1038/s41398-021-01713-z (PMC8590689; doi:10.1038/s41398-021-01713-z)
Supplement: Supplementary file 5 — Supplementary Table 5 [file 41398_2021_1713_MOESM5_ESM.docx]

**Supplementary Table 5**: Estimates of SNP heritability of Cognitive Performance (CP), Education Attainment (EA), Household Income (HI), General Risk-taking behavior (GR) and Psychopathology(PGC_CD) based on LD score regression.
